# Empowering promotoras through community-based participatory research in Latinx and Indigenous Mexican communities during the COVID-19 pandemic

**DOI:** 10.3389/fpubh.2025.1655892

**Published:** 2026-01-08

**Authors:** Ann Marie Cheney, Erica Li, Gabriela Ortiz, Alexa Pazos, Carina Bell, Evelyn Vázquez, Luis Delgado, Arianna Zimmer, Úrsula Simonoski, Rocio Muñante Navarro, Maria Pozar

**Affiliations:** 1 Department of Social Medicine, Population, and Public Health, School of Medicine, University of California, Riverside, Riverside, CA, United States; 2 School of Medicine, University of California, Riverside, Riverside, CA, United States; 3 Department of Anthropology, College of Arts, Humanities, and Social Sciences, University of California, Riverside, Riverside, CA, United States; 4 Internal Medicine – Primary Care Track, Cedars-Sinai Medical Center, Los Angeles, CA, United States; 5 Department of Pediatrics, School of Medicine, University of California, Irvine, Irvine, CA, United States; 6 Program of Public Health, University of California, Irvine, Irvine, CA, United States; 7 Family Medicine, Eisenhower Medical Center, Rancho Mirage, CA, United States; 8 Charles R. Drew University of Medicine and Science, Willowbrook, CA, United States; 9 Conchita Servicios de la Comunidad, Mecca, CA, United States

**Keywords:** COVID-19 health inequities, community based participatory research, community capacity building, COVID-19 pandemic response, Latinx immigrant farmworkers, Indigenous Mexican immigrants, trust building, community health workers and promotoras as researchers

## Abstract

**Introduction:**

This study describes an innovative community-based participatory research model that engages community health workers/promotoras as research partners to address health inequities among Latinx and Indigenous Mexican immigrant communities during the COVID-19 pandemic.

**Methods:**

Using ethnographic and qualitative research methods, we collaborated with promotoras trained in qualitative data collection and analysis to conduct seven focus groups (six in Spanish, one in Purépecha) with 55 participants in a rural desert region of Inland Southern California. Rapid analytic techniques were applied to identify emergent themes across data sources.

**Results:**

Findings revealed deep-seated mistrust of institutions in the United States, including government, healthcare, and public health, fueling fear and suspicion around COVID-19 testing and vaccination. Promotoras bridged communities and institutions through their dual roles in public health response (contact tracing, testing and vaccination clinics, and health education) and participatory research (data collection, analysis, dissemination).

**Discussion:**

This capacity-building approach positioned promotoras as trusted leaders such as science and public health education, demonstrating that engaging promotoras as co-researchers can strengthen institutional trust, enhance community participation, and advance health equity.

## Introduction

Latinx populations in the United States (US) were disproportionately affected by the COVID-19 pandemic. Structural and social determinants of health, including poverty, food and housing insecurity, limited English proficiency, fear of deportation, and restricted access to healthcare, heightened vulnerability to viral transmission, morbidity, and mortality (1, 2). Historical and ongoing mistrust in science, government institutions, and public health campaigns further shaped attitudes and behaviors around COVID-19 testing, vaccination, and healthcare utilization, thereby contributing to elevated transmission, hospitalization, and mortality rates among Latinx communities (3–6).

One evidence-based approach to improving health literacy and rebuilding trust in healthcare and public health systems is through the engagement of community health workers (CHWs), or promotoras—community leaders and laypersons who receive specialized training to serve as cultural and linguistic brokers between communities and healthcare systems (7, 8). We intentionally use the gendered term ‘promotoras’ to recognize the central role of women in preventive health and community-based interventions during the COVID-19 pandemic. Across Latinx and Indigenous Mexican immigrant communities, women have historically taken on caregiving and health advocacy roles within families and neighborhoods, positing them as trusted messengers and organizers (9). The promotor model, which began in Latin America in the 1950s and trained women (promotoras) to share health resources with those in rural and marginalized communities, and is deeply rooted in cultural traditions of mutual aid and collective responsibility (10).

CHWs/promotoras have demonstrated effectiveness in improving health knowledge and promoting behavioral changes related to chronic disease prevention (11). As trusted messengers and advocates, they played a pivotal role in mitigating the impacts of the COVID-19 pandemic by promoting testing and vaccination, countering misinformation and disinformation, facilitating digital engagement, and addressing mental health needs (12–14). Recognizing promotoras as both gendered and community-embedded leaders underscores their unique ability to bridge cultural, linguistic, and institutional divides while advancing health equity in Latinx and Indigenous Mexican populations (8).

Addressing these disparities requires community-driven strategies that bridge the gap between public health systems and marginalized populations. As such, the goal of this manuscript is to present an innovative model for engaging promotoras in health disparities research through community-based participatory research (CBPR). Specifically, we describe how promotoras collaborated with academic researchers, healthcare institutions, and public health agencies during the COVID-19 to mitigate its impact on vulnerable populations. Our work focuses on research and public health efforts addressing the health and social needs of Latinx and Indigenous Mexican farm working communities—groups disproportionately affected by the COVID-19 due to high rates of existing chronic health conditions and limited social and employment protections (15–18). We highlight the critical role of promotoras as trusted community leaders in bridging relationships between marginalized communities and institutional systems that have historically fostered mistrust, fear, and skepticism toward public health measures (4). Grounded in the principles of CBPR, our model operationalizes CHW/promotora engagement as both a research method and a mechanism for advancing health equity in structurally marginalized communities.

## Materials and methods

### Setting

This project was carried out from 2020 to 2021 in the Eastern Coachella Valley (ECV) of Inland Southern California, which is home to Latinx and Indigenous Mexican immigrants of low income who work in the nearby agricultural fields. The ECV has some of the richest agricultural lands in California, and farmworkers in this region contribute significantly to the American food system. This population is vulnerable to inequities in health due to their low social status (e.g., non-US citizens, undocumented status), precarious working conditions, low-wage farm labor, and limited access to healthcare services due to geographic (rurality) and cultural (language) barriers (19). The ECV is also home to the largest state-side community of Purépecha, an indigenous group from the state of Michoacán, Mexico. Approximately 6,000 to 10,000 members of this community, most of whom are from Ocumicho, live in this rural desert region (20).

In the early months of the pandemic, this region was a hotspot for COVID-19 transmission with an infection rate five times higher than other communities in the valley (21).

### Project team and capacity building

This project employed principles of CBPR, a collaborative approach that draws on the expertise of community and academic partners to promote shared decision making, equitable allocation of resources, and the co-creation of knowledge (22). The Principal Investigator, a PhD-trained anthropologist and health services researcher, and the Community Investigator, a Latina immigrant and member of the Purépecha community, co-led the project. Together, they oversaw a trilingual team (English, Spanish, Purépecha), comprised of a Co-Investigator (PhD-level Latina community psychologist), six Latina promotoras, seven medical students, two graduate students, and three undergraduate students.

The team’s COVID-19 response emerged from pre-existing collaborations. One year prior to the pandemic, the community and academic leads had partnered with a federally qualified healthcare system and medical students and clinical faculty to establish a free primary care clinic serving Spanish- and Purépecha-speaking farmworker communities in the ECV. Because of this established trust and reputation for effectively engaging vulnerable populations, particularly low income, limited-English-proficient farmworkers, a local foundation invited the academic lead to extend these partnerships to establish COVID-19 testing sites for farmworkers and their families (23). In response, the community and academic partners collaborated with their healthcare and public health partners to co-design culturally and linguistically appropriate COVID-19 testing sites and to disseminate accurate public health education. The community academic partnership team evaluated the impact of these efforts on perceptions of the virus, transmission, and attitudes and behaviors regarding testing and future vaccination.

To guide these efforts, a 12-member Community Advisory Board (CAB) was convened, composed of community leaders, promotoras, physicians, medical students, healthcare system representatives, public health partners, and growers to engage diverse voices and multiple perspectives in project activity. The CAB meet four times between September to December 2020 for 90 min per session. Members provided input on testing site logistics, engagement strategies for vulnerable Latinx populations and data collection methods, including survey design, focus group topics, and dissemination plans.

Our community-academic team held weekly meetings throughout the project period to collectively make decisions about project and research activity and engagement of vulnerable communities in COVID-19 public health measures. As part of our innovative model, we prioritized research capacity-building among promotoras and students through a 4-h structured training that emphasized co-learning and skill development in qualitative research. Prior to the training sessions, participants reviewed readings and materials on qualitative data collection and analysis.

The first training session emphasized co-learning in qualitative research methods, focusing on data collection through focus groups and semi-structured interviews (see Supplementary material for the reading guide). Team members, comprising promotoras, students, and academic partners—completed preassigned readings and used a reading guide with reflective prompts such as “When would you conduct a focus group?” and “What are some advantages and disadvantages of focus groups?” to foster shared discussion and critical thinking. The training also included information on recruitment with a focus on snowball sampling.

The second training sessions built on this foundation by introducing collaborative approaches to qualitative data analysis. Participants learned how to transform recorded interviews into transcripts and engage in systematic, team-based analysis to identify key themes. The training emphasized developing and applying structured templates aligned with interview guide domains to summarize participant responses. These summaries were then organized into a matrix to facilitate collective interpretation and comparison across focus groups. This iterative participatory process enhanced both methodological rigor and shared ownership of the research findings.

The lead author (AMC) applied this approach as part of an anti-oppressive, decolonial framework for qualitative inquiry, drawing on prior collaborative research that centered Indigenous knowledge and community wisdom in data collection, analysis, and interpretation (24). The skills developed through this training were subsequently applied in the conduct and dissemination of community-driven public health research. Promotoras and students collaboratively collected, analyzed, and interpreted focused group data, using their findings and lived observations to co-develop culturally grounded COVID-19 public health educational materials.

### Data collection

The study was carried out from August 2020 to February 2021. Ethnographic and qualitative research methods were used to better understand the context of COVID-19 in Latinx and Indigenous Mexican immigrant farm-working communities and elicit shared experiences, and a brief socio-demographic survey collected participants’ characteristics. Prior to the start of data collection, ethical approval was obtained from the University of California Riverside Institutional Review Board, protocol HS-17-187. This research aligns with the world medical association declaration of Helsinki-ethical principles for health research with human subjects. For ethnographic data collection, team members indicated their role as a member of a research team investigating COVID-19 health disparities during community meetings and interactions and obtained verbal consent documenting this in notes. Prior to the start of qualitative data collection (i.e., focus groups), promotoras administered the consent form to participants who provided verbal consent and recorded this in the Qualtrics survey platform. Because this research was carried out virtually (phone and Zoom video conferencing) during the COVID-19 pandemic, written informed consent was not possible, and the IRB approved verbal consent.

#### Ethnography

Ethnographic methods were used to contextualize community and institutional dynamics shaping pandemic response efforts in Latinx and Indigenous Mexican farm working communities. Observations were conducted during monthly CAB meetings (n = 4), virtual conversations with growers and farm laborer employers, public meetings related to agricultural health and safety, and conversations with healthcare system and public health leaders during the initial months of the pandemic. While the academic partners led the ethnographic components of the study, promotoras contributed detailed observations of COVID-19 testing sites and community interactions, drawing from their lived experience and trusted relationships with residents. These observations were systematically incorporated into field notes.

#### Focus groups

A total of seven focus groups were conducted in November and December 2020, six in Spanish and one in Purépecha, with community members from farm-working communities in the ECV. Our approach to data collection aligned with recommendations for collecting and analyzing focus group data in which 90% of themes or patterns can be identified within three to six focus groups with nonprobability and homogenous (e.g., low-income, Latinx, geographically similar) samples (25). Zoom Video conferencing was used to hold the focus group sessions and record them.

#### Recruitment and focus group eligibility

We used purposive (nonrandom) sampling to recruit participants into the study (26). In teams of two, promotoras shared the study flyer throughout their social networks to recruit participants to assist in a focus group set for a predetermined day and time that they would facilitate. Each team was asked to recruit 12 participants to account for attrition and ensure at least 6–8 participants. Participants were eligible if they: (1) were 18 years of age or older, (2) self-identified as Latinx/Hispanic and/or Purépecha, (3) lived in the ECV (i.e., Coachella, Thermal, Mecca, Oasis, North Shore), and (4) spoke Spanish or Purépecha. All focus groups were held the same day and time. Using the Zoom breakout rooms function, we placed participants into their assigned focus groups (organized by promotora team) and used Zoom to record each focus group which lasted from 60 to 90-min. For participants unable to access the Zoom video conference platform, we provided the Zoom conference line information using one-touch mobile access. In cases when participants were unable to access either the Zoom platform or conference line, a team member called the participant using their phone and placed the participant on speaker phone so they could participate in the discussion. Focus groups ranged between 75 to 90 min. Participants received a $20 gift card for their time. At the close of the focus groups, participants completed a brief anonymous survey, either self-administered, via online survey, or interview administered by a team member via phone (Table 1).

**Table 1:** Semi-structured interview guide.

Topic	Question	Probes
Coronavirus	Let us start by talking about the coronavirus. What do people in your communities think about the virus?	How do people understand how the virus spreads? What kind of confusion is there about the virus?
	Now, let us talk about virus-related behaviors. Can you tell us how people in your communities are trying to reduce the spread of the virus?	Do people in your community wear masks? What does social distancing mean in your community? Do people get together? Why or why not?
	What can we do to increase behaviors that help slow the spread of the virus in your communities?	
COVID-19 testing	From what you have heard, what are people’s thoughts on COVID-19 testing?	Why would people in your community get tested? What are some barriers or fears that can make it difficult for people in your communities to get tested? What might people in your community think about public health, government, or health care providers? Do they trust them? Why or why not?
	What can we do to help people in your community who test positive for COVID-19?	What kind of resources and support do people need? Such as help with finances, mental health, or employment.
COVID-19 vaccine	Now, let us talk about the COVID-19 vaccine. What do people in your community think about the COVID-19 vaccine?	What are some things that might make it harder or easier for people to get the vaccine?
	Once the vaccine is available, do you think people will get the vaccine?	What could be done in your community to improve what people believe about the COVID-19 vaccine? What strategies, information, or resources are needed?

Promotoras ran the focus groups with the assistance of medical and undergraduate students who helped by co-facilitating the sessions and taking notes. The semi-structured interview guide included questions on the following topics: community beliefs and attitudes about the coronavirus, its spread, COVID-19 testing, and adoption of COVID-19 risk-reduction behaviors such as mask wearing, social distancing, group gatherings. (See Table 1 for semi-structured interview guide). Facilitators used follow-up probes to elicit additional information about public health outreach, testing events, and strategies to support those with COVID-19.

### Qualitative data analysis

Ethnographic and qualitative data were analyzed using two complementary rapid qualitative coding techniques: summary templates and matrix analysis (27, 28). This technique includes both deductive and inductive theme identification. Thus, the codebook includes *a priori* codes based on the interview guide (e.g., community perceptions of the virus, COVID-19 testing and vaccination) and emergent themes, including fear, mistrust, and role of promotoras in the pandemic response, based on an inductive analysis. Audio recordings were transcribed, verified for accuracy, and reviewed collaboratively by promotoras, students, and academic researchers under the guidance of qualitative experts (AMC, EV). Reading line by line, the team summarized participant responses, recorded analytic reflections, and identified illustrative quotes. The resulting summaries were organized by domain from the interview guide and included sections for analytic memos and exemplar quotes.

**Figure 1:**
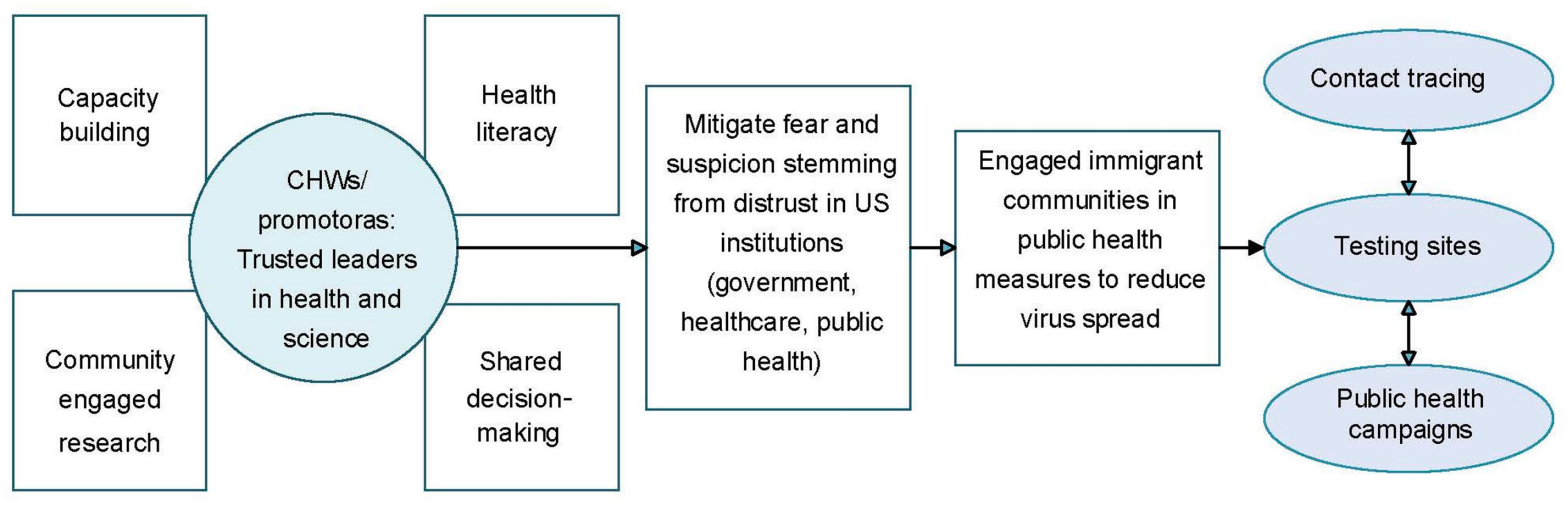
Role of community health workers/promotors in mitigating distrust in US institutions.

To facilitate cross-group analysis, the team constructed a matrix (focus group X domain/interview guide question) using a Google Sheet in which summarized data were condensed into short, descriptive phrases (e.g., “concerned about vaccine development speed”). This allowed for systematic identification of recurring themes and cross-cutting patterns. Through iterative team discussions and collective interpretation sessions, promotoras played a central role in contextualizing findings, drawing on their on-the-ground insights to refine thematic interpretations and shed light on collective experiences and community perspectives. This iterative process resulted in the co-development of a theoretical model illustrating relationships among key themes (see Figure 1). Exemplar quotes were selected to ground the model in participants’ voices (29, 30).

Throughout the analytic process, promotora’s ethnographic contributions and interpretative insights were critical to ensuring cultural validity and community relevance. Team meetings included reflection in which promotoras and academic partners discussed how the findings reflected lived community realities and perspectives, producing memos that deepened interpretation through a community-engaged, equity-centered lens.

## Results

### Promotoras as partners in the pandemic response and research

All six promotoras, were Latina women residing in the ECV. One worked as a farm laborer, offering firsthand understanding of farm working conditions and health challenges. All had prior experience serving as promotoras in the context of community and public health, and two had collaborated with the community-academic team on earlier research initiatives. Spanish was their primary language. Two had bachelor’s degrees whereas others had completed primary or middle school. Collectively, the promotoras brought extensive local knowledge, linguistic and cultural competence, and trusted relationships within their communities, which positioned them as essential partners in both data collection and interpretation. Their lived experiences and community leadership informed the study’s design, analysis, and contextual understanding of pandemic-related health inequities.

Promotoras played a pivotal role in restoring community trust and facilitating access to public health resources and services. In partnership with public health, healthcare systems, and community advocacy groups, they sought accurate information on COVID-19, employment protections, and testing resources. They staffed contract-tracing programs, assisted at testing clinics, distributed food boxes and educational materials, and hosted Spanish- and Purépecha-language online and in-person talks. Two Spanish-speaking promotoras from communities in the ECV and a bilingual (English/Spanish) premedical student who had worked in the community were employed by the department of public health. They were hired and trained to work directly with patients in the ECV who tested positive for COVID-19, or businesses and organizations that had positive cases of COVID-19 among their employees.

From September to December 2020, promotoras participated in 26 testing clinics serving approximately 1,470 community members of their communities. They were often the first to greet patients at testing clinics and provide food boxes and resources as patients exited. This approach aligned with what we observed and heard from community both at testing sites and focus groups:

There are people who simply do not have transportation to go and get a test for COVID. . . . [T]here are still people who do not like to ask for help, [but] they really need it [information]. Well, to have like [access to] resources, right, help for the transportation.

Promotoras attended weekly health equity collaborative meetings with public health officials to obtain up-to-date information on virus spread and testing locations. They disseminated this and other up-to-date, accurate public health information during Facebook Live COVID-19 talks, or pláticas in which they collaborated with healthcare professionals (medical students, healthcare providers, therapists) to disseminate information and respond to community concerns. They also collaborated with a bilingual (Spanish/English) medical doctor from the ECV who had served as a farm worker in the fields as a teenager. In total, they hosted 22 online talks reaching 29,657 unique viewers and 10 in-person talks with 80 community members. They also created a list of over 400 community members who received weekly text messages with testing site locations and hours.

Additionally, the promotoras played a pivotal role in conducting the community-based COVID-19 health disparities research. Drawing on their trusted relationships and linguistic and cultural fluency, they led recruitment efforts, engaging 55 community members from the ECV to participate in focus groups. The promotoras facilitated seven focus groups—held in Spanish and Purépecha—creating safe and linguistically accessible spaces for participants to share their experiences of the pandemic. In addition to facilitating discussions, they also consented participants, assisted in survey administration, and contributed ethnographic observations.

A total of 55 community members participated in the focus groups, and 53 completed the socio-demographic survey. As shown in Table 2, most participants were female (81%) and over half (51%) were between 45 and 64 years old. All identified as Latino or Indigenous Mexican with 83% identifying as Hispanic or Latino and 17% as Purépecha. Most participants were employed full- or part-time (53%), over a third (36%) identified as a farmworkers, and a majority (56%) lived in the unincorporated agricultural communities of Oasis, Thermal, and Mecca. Over three-fourths (83%) reported being affected by COVID-19 and 86% expressed fear the virus and its spread.

**Table 2:** Focus group participants’ characteristics – 2020 research conducted in Inland Southern California.

Characteristic	***N* = 53 (%)**
Gender	
Female	43 (81)
Male	10 (19)
Ethnicity/race	
Latino/Hispanic	44 (83)
Purépecha	9 (17)
Age	
18–24	5 (9)
25–34	21 (40)
45–54	23 (43)
55–64	4 (8)
Community	
Unincorporated (Mecca, Thermal, Oasis)	30 (56)
Salton City	3 (6)
Indio	7 (13)
Coachella	11 (21)
Employment status	
Full time (40 h/week)	10 (19)
Part time (less than 40 h/week)	18 (34)
Stay at home caregiver	8 (15)
Student	2 (4)
Unemployed or disabled	15 (29)
Farm laborer	19 (36)
Accessed Benefits (yes)	45 (85)
Health insurance (yes)	38 (72)
Have you felt affected by the coronoavirus?	44 (83)
Are you afraid of the coronavirus?	
Not scared	6 (12)
Scared	46 (86)

* Of the total number of focus group participants (n = 55), 53 completed the socio-demographic survey. Percents may not add up to 100% due to missing data.

Through their leadership in recruitment, facilitation, and data collection, the promotoras advanced a community-driven research process that centered local knowledge and strengthened the study’s cultural and linguistic relevance amidst the pandemic.

### COVID-19 context in Latinx and Indigenous Mexican immigrant communities

Our findings reveal a pervasive distrust of US institutions, including government, healthcare, and public health systems among Latinx and Indigenous Mexican immigrant communities in the ECV. This distrust fostered fear, uncertainty, and suspicion that shaped decisions regarding COVID-19 testing, vaccination, and healthcare utilization.

As trusted community members and research partners, the promotoras played a pivotal role in mediating this distrust. Their dual engagement in community-driven research and public health initiatives positioned them as cultural and linguistic bridges between residents and formal institutions. Drawing from their lived experience and deep community relationships, promotoras led culturally responsive outreach and education, facilitated focus group discussions, and interpreted community narratives that highlighted the structural and historical factors underpinning mistrust.

Their leadership in contact tracing, testing clinics, and health promotion efforts improved access to information and services and helped re-establish channels of trust between historically disadvantaged communities and institutions (see Figure 1). This collaborative approach not only mitigated community mistrust and enhanced participation in COVID-19 prevention efforts, but also advanced promotora’s research and leadership skills, laying the groundwork for sustainable, community-driven infrastructure to address ongoing health inequities.

#### Distrust and suspicion

Participants described widespread distrust and suspicion toward US institutions, including the government, healthcare, and public health systems, rooted in long-standing experiences of marginalization and intensified by the uncertainties of the pandemic. Many viewed institutional responses to COVID-19 through the lens of historical neglect and systemic inequity. Some participants expressed beliefs that the virus was intentionally created by the US government to harm vulnerable populations such as older adults and immigrants. “I heard at work that the virus, in China it’s spread, all of this, it’s because the [US] government wanted to get rid of all older adults people,” a participant said.

Several participants also viewed the vaccine rollout as experimental or exploitative, reflecting broader fears of being used as test subjects or excluded from equitable healthcare. A participant shared:

Here in the desert, they [US government] are more so experimenting. I would not take my children nor myself to get this “amazing vaccine” . . . I am only saying they are just experimenting. It [the vaccine] is useless.

Promotoas, who facilitated the focus groups and interpreted these narratives, contextualized such perspectives as stemming from a long history of medical and government mistrust in marginalized immigrant communities. In this case, the narrative underscores how rurality and geographic isolation increased vulnerability to institutional abuse. Their engagement in data collection and interpretation helped identify how structural inequities and misinformation intersected to produce skepticism toward public health efforts. As the following quote illustrates, this skepticism eroded confidence in vaccine development:

“It’s crazy medicine. There must be years of follow up to know what consequences the vaccine has before it’s put on the market. The vaccine will come out too quickly for us to know the consequences it’ll have.”

Participants associated healthcare systems with harm and death. One participant commented: “I have heard many people who went [to the hospital] for one thing and suddenly they have COVID, that everyone was leaving the hospital dead from COVID.” One participant recounted her neighbor’s story:

He was calling them [his family] and was telling them what he was afraid of, that there were things happening, ugly things that were happening to him. His daughter stopped going to the hospital . . . because they [hospital staff] did not let them [the family] have contact with him at all. . . . But before he passed away, he had called his daughter to ask her to come and get him because he didn't feel well there.

Another shared that hospitals were misreporting deaths to benefit financially:

I have also known of many people [who died]. There was one person that died of a heart attack and they [hospital staff] were saying that it was COVID. They [hospital staff] wanted the person to sign their relative’s letter, accept that they had died of COVID when the person had died from a heart attack. The question here was because if he died from COVID they cremate you, right? And they don’t let you see the body . . . it’s a fight with the healthcare system to take the person’s body . . . it’s because it suits them [the healthcare system] the people who die, die of other things but they say it’s the coronavirus to get more resources from the government. I wouldn’t go to the hospital, it’s a questionable place.

#### Fear

Fear of COVID-19 testing, job loss, and deportation discouraged preventative behaviors. Many described avoiding testing because a positive result could lead to lost wages, employment termination, or unwanted scrutiny of immigration status. As one participant explained: “I know people who are afraid to go [get tested], and that they will become positive [for COVID-19] and these are people who work in the fields.” Economic precarity was a pervasive theme, as participants emphasized that many farm workers are undocumented and lack access to worker’s compensations or paid sick leave. “There is always that fear of being undocumented” another participant shared when describing hesitation to seek testing or healthcare.

Promotoras, who facilitated these discussions and contextualized participants’ experiences, described fear as both a rational response to structural vulnerability and a barrier to public health engagement. Their insights helped connect individual expressions of fear to broader social determinants such as employment insecurity, immigration status, and limited healthcare access that intensified the pandemic’s impact on immigrant farmworker communities. It was their dual roles as trusted community members and researchers and insight that shed light on how fear operated as an emotional response and reflection of systemic inequity and precarity.

Many pointed out the need for public health information and employment and mental health resources at testing sites as a method to reduce fear and support those who tested positive:

Financial support, help with their work, in case that they are positive. . . What processes do they have to carry out their work, so they are not fired, so that they are allowed that time off, so that they get paid while they’re sick.

Maybe at a testing site there could also be information or resource, in case you test positive, you can go and ask for help, such as psychological help, economic help, employment help. In case you test positive: What process do you need to move forward in your job, so they do not fire you? That they let you take leave and pay you while you are sick.

As these quotes illustrate, participants’ reflections highlight the need for a pandemic response that considered basic needs as well as emotional and economic wellbeing. Through their facilitation and interpretation of these discussions, promotoras helped reveal how fear and distrust were rooted in misinformation and structural vulnerabilities.

## Discussion

Our work highlights the value of CBPR in addressing health disparities during the COVID-19 pandemic and demonstrates the critical role of promotoras as co-researchers and trusted community leaders. Through their engagement, our team was able to document how distrust and suspicion, fear, and structural vulnerability shaped pandemic experiences among Latinx and Indigenous Mexican immigrants in rural Inland Southern California. These findings mirror those of others who found that immigrant and low-income, often deemed “essential labor” faced heightened risk of exposure due to overcrowded and substandard housing conditions, limited protections, and restrictive public policies such as the public charge rule (31).

Consistent with prior studies, our findings show that promotoras served as “puentes”—bridges linking vulnerable communities with healthcare and public health systems (10, 32). Beyond facilitating recruitment into research, they led data collection and co-led data analysis and dissemination expanding the traditional role of CHW involvement in research. Bloss et al.’s (33) mapping review identified a lack of empirical studies in which CHWs/promotoras contributed to data collection, analysis or dissemination. Thus, our model addresses this gap offering insight into participatory data collection and analytic methods to engage CHWs in all stages of the research process.

Because of their lived experiences and positionality within their communities, the promotoras were uniquely positioned to meaningfully interpret study findings contextualizing fear of testing and vaccination within broader socioeconomic and legal precarity (19). They understood the underlying socioeconomic reality of farm workers and other vulnerable groups (e.g., Purépecha speakers) in their communities. The promotoras connected farmworkers and other vulnerable community members to testing services, financial and legal resources, and basic needs, as well as communicated lived realities of others in their community with public health officials and leadership in healthcare systems. They translated what they learned through research into immediate advocacy. These findings echo Saldanha’s (18) ethnographic work among migrant farmworkers in Michigan showing that outreach workers serve as trusted brokers who connect migrant laborers and vulnerable groups to supportive and health-promoting services.

Our study calls for promotoras to be recognized not only as outreach workers (e.g., see (34)) but also as co-producers of knowledge in health equity research. Their involvement advanced both the quality and impact of our research, creating an evidence base for real-time public health action. This collaborative, cross-sector model also informed subsequent community-engaged COVID-19 research with multiple health disparity populations in Inland Southern California contributing to National Institutes of Health California-wide Community Engagement Alliance Initiative (CEAL) initiative (35).

Furthermore, our findings underscore the gendered nature of community health work. The promotoras on our team—like others in Latino communities throughout the US—bore the emotional and logistical burdens of serving their communities as first responders during the pandemic (12, 32). Like members of their communities, they too had to navigate the same fears and economic and political uncertainty. Their roles involved constant emotional labor in which they offered empathy and support to others while managing their own anxiety (36). Acknowledging the gendered nature of this role highlights the need for structural level change such as stable employment, fair pay and benefits, training and certification, and access to preventive health care and mental healthcare services—the latter of which is essential during public health emergencies. As the pandemic taught us, investment in CHW/promotor programs is critical for public health preparedness and equity. Rather than rely on short-term contracts during crises—which is what happened during the pandemic—local and state health departments should institutionalize these positions for CHWs/promotoras so they become part of community-level healthcare infrastructures.

### Limitations

This study offers important insight on the role of promotoras in mitigating distrust in immigrant communities during the early months of the COVID-19 pandemics. However, several limitations should be considered when interpreting the findings. First, this study was conducted prior to the large-scale rollout of the COVID-19 vaccine in the US in winter 2021. At the time of data collection (November to December 2020), the vaccine had not yet been approved by the Food and Drug Administration (FDA). Thus, participant discussions about the vaccine reflected the uncertainty about its safety, availability and development stage.

Second, we did not conduct individual interviews with promotoras who were part of the research team. As a result, our data capture their community-facing roles and observed actions but not a systematic documentation of their personal experiences, reflections, or challenges navigating their role and being a member of the research team. Such interviews could have provided deeper understanding of the gendered and emotional dimensions of promotor work especially the emotional toll of serving as bridges/puentes in communities hit hard by the pandemic (36). Future research should center the voices of promotoras documenting their professional and gendered identity and their emotional labor. Such work would deepen understanding of CHW/promotor-led public health initiatives and inform workforce policies to ensure adequate compensation, mental health support, and institutional acknowledgement (37).

Third, participation in the focus groups required access to the internet, an electronic device such as laptop, smart phone, or tablet, and a private or semi-private space. It is possible that more vulnerable community members, meaning those without such technologies or space within their living environments, were not able to participate in the study. To mitigate this limitation, we provided participants with the access the Zoom conference line to access the focus group discussion. However, because the Zoom line has English instructions on how to enter the meeting identification number, many who intended to participate via the phone were unable to access the Zoom space.

Last, the COVID-19 pandemic is no longer a public health emergency in the US or globally limiting the public health impact of study findings. Despite this limitation, our work offers important insight into the role of promotoras as members of community-academic teams involved in the research lifecycle.

## Conclusion and future research

Fear and suspicion linked to a history of institutional distrust underpinned decisions around access to COVID-19 related healthcare services. For Latinx and Indigenous Mexican immigrant communities in Southern California, racialized labor conditions, socio-economic precarity, and immigration status intersected to shape access and perceived safety in seeking COVID-19 testing and vaccination (2, 4, 38). These findings illustrate how structural inequities, rather than individual hesitancy, framed COVID-19 health-related decisions.

Our study underscores that trust in public health is dynamic. Promotoras play an important role in building relationships between structurally vulnerable communities and health systems. Their positionality within their communities permits them to not only effectively communicate public health information and dispel misinformation but also translate research findings into meaningfully and immediate public health action.

## Data Availability

The raw data supporting the conclusions of this article will be made available by the authors, without undue reservation.
