# Supplementary material for: Empowering promotoras through community-based participatory research in Latinx and Indigenous Mexican communities during the COVID-19 pandemic
Source: Front Public Health. 2026 Jan 8;13:1655892. doi: 10.3389/fpubh.2025.1655892 (PMC12823809; doi:10.3389/fpubh.2025.1655892)
Supplement: Supplementary file 1 [file Data_Sheet_1.PDF]

Training: Qualitative Data Collection and Analysis

Reading Guide

This guide will help you read the readings that are part of the qualitative data collection and analysis training. Below are some key points and questions that will guide you. Please provide a brief response to each question.

Assigned readings:

- Focus groups (Grupos de Enfoque). UCLA Center for Health Policy Research. Health DATA—Datos. Abogacía. Entrenamiento. Asistencia.
- Conducting interviews. University of Kansas Community Toolbox. Available in Spanish: https://ctb.ku.edu/es/tabla-de-contenidos/valoracion/valorar-las-necesidades-y-recursos-comunitarios/conducir-entrevistas/principal
- Focus groups and content analysis in qualitative research: Silveira Donaduzzi, Daiany Saldanha da et al. Grupo focal y análisis de contenido en investigación cualitativa. *Index Enferm* [online]. 2015, vol.24, n.1-2, pp.71-75. ISSN 1699-5988. doi.org/10.4321/S1132-12962015000100016.

<u>What is a focus group?</u>

A focus group is a group interview. The ideal group size is 6 people. We typically do at least 2 or 3 focus groups in a single study to obtain in-depth responses to answer research questions. Focus groups typically last between 60 or 90 minutes and are led by a facilitator.

<u>Questions to consider when reading the assigned articles:</u>

1. When would a focus group be conducted?
2. When selecting focus group participants, what factors are important? (e.g. must have similarities, etc.)

3. What are some advantages and disadvantages of focus groups?
4. One technique for selecting focus group participants includes 'snowballing'. What is this technique?
5. An interview instrument typically has 5 parts. What are these parts?
    a. Part 1:
    b. Part 2:
    c. Part 3:
    d. Part 4:
    e. Part 5:
6. What is the role of the moderator (or facilitator) in a focus group? What are some characteristics of an ideal moderator?
7. What are some techniques for moderating the focus group? (e.g., pause and test, don't let one person dominate the discussion)
8. How can you ensure the confidentiality of participants in a focus group?

How do you conduct a qualitative interview?

It is the responsibility of the interviewer to create a relationship with the people who will be interviewed. When interviewing someone, begin with small talk: "How are you?"; "Did you have any problems getting into the Zoom space?" It’s also the responsibility of the interviewer to guide the conversation and finish on time.

Reading

<u>Questions to consider when reading the assigned articles:</u>

1. What are some points to remember? (e.g., active listening)

2. How can facilitators/interviewers ask questions in a way that motivates the interviewee to answer the questions in detail and depth?
3. Why is it important to avoid judgmental questions such as: "Why did you do something so bad?
